# Comparative cytogenetics of Neotropical cichlid fishes (*Nannacara*, *Ivanacara* and *Cleithracara*) indicates evolutionary reduction of diploid chromosome numbers

**DOI:** 10.3897/CompCytogen.v8i3.7279

**Published:** 2014-08-08

**Authors:** Lucie Hodaňová, Lukáš Kalous, Zuzana Musilová

**Affiliations:** 1 Department of Zoology and Fisheries, Faculty of Agrobiology, Food and Natural Resources, Czech University of Life Sciences Prague, Prague, Czech Republic; 2 Laboratory of Fish Genetics, Institute of Animal Physiology and Genetics AV CR, Libechov, Czech Republic; 3 Zoological Institute, University of Basel, Switzerland

**Keywords:** Cichlid cytotaxonomy, cyt b, 16S rRNA, S7-1, RAG1 phylogeny, karyotype differentiation, CMA_3_ phenotypes, Cichlasomatini

## Abstract

A comparative cytogenetic analysis was carried out in five species of a monophyletic clade of neotropical Cichlasomatine cichlids, namely *Cleithracara maronii* Steindachner, 1881, *Ivanacara adoketa* (Kullander & Prada-Pedreros, 1993), *Nannacara anomala* Regan, 1905, *N. aureocephalus* Allgayer, 1983 and *N. taenia* Regan, 1912. Karyotypes and other chromosomal characteristics were revealed by CDD banding and mapped onto the phylogenetic hypothesis based on molecular analyses of four genes, namely cyt *b*, 16S rRNA, S7 and RAG1. The diploid numbers of chromosomes ranged from 44 to 50, karyotypes were composed predominantly of monoarmed chromosomes and one to three pairs of CMA_3_ signal were observed. The results showed evolutionary reduction in this monophyletic clade and the cytogenetic mechanisms (fissions/fusions) were hypothesized and discussed.

## Introduction

Cichlids are a species-rich group of ray-finned fishes (Actinopterygii), distributed in tropical and subtropical freshwaters of Africa and South and Central America, Texas, Madagascar, the Middle East, India and Sri Lanka (Kullander 1998). As a third largest fish family (Eschmeyer and Fricke 2012) cichlids represent highly evolutionarily successful fish lineage and it is considered that no other family of vertebrates exceeds cichlids in a number of varieties, shapes, colors and especially in ecological and trophic specializations (Kocher 2004).

In general, genomes of ray-finned fishes are known for high evolutionary dynamics among vertebrates, which is reflected in huge genome-architecture variability (Mank and Avise 2006). The diploid chromosome number (2n) studied in 615 Actinopterygian species ranges from 22 to 250, but over a half of the species possess the conservative number of 2n = 48 – 50 chromosomes (29.3% have 2n = 48 and 25.4% have 2n = 50; Mank and Avise 2006). The most frequent fish karyotype, i.e. 2n = 48 (n=24), is also recognized as an ancestral karyotype of the whole Teleostei (Ohno et al. 1969, Nakatani et al. 2007).

In total, over 190 cichlid species have been cytogenetically analyzed and the karyotype formula was determined for 157 of them (Arai 2011). Available cytogenetic data in cichlids show that the diploid chromosome numbers range from 2n=32 to 2n=60, but more than 60% of the examined species show the ancestral karyotype with 2n=48, which mostly dominates in the Neotropical cichlid lineage (Feldberg et al. 2003).

In the past only few species were analyzed and Neotropical cichlids were considered a karyotypically conservative group due to the frequent findings of 48 chromosomes (Thompson 1979, Kornfield 1984). Later, Marescalchi (2004) and Poletto et al. (2010)demonstrated much higher variability in the chromosome number and hypothesized that the ancestral karyotype of the Neotropical cichlids underwent significant changes in structure in several lineages, which led to extensive karyotype diversification. Further, many species possess the similar 2n=48, but differ in karyotype structures, which brings additional evidence of the karyotype differentiation due to the intra-chromosomal rearrangements like centromeric shifts (Feldberg et al. 2003). It is likely that at least some different lineages coincidentally converged to the same number of chromosomes from different ancestral stages but the mechanisms of why there is certain favorable number of chromosomes remains still unknown (Mank and Avise 2006).

Dwarf cichlids of the genus *Nannacara* Regan, 1905, and its relatives, genera *Ivanacara* Römer & Hahn, 2007 and *Cleithracara* Kullander & Nijssen, 1989 represent a well-defined evolutionary lineage of acaras (NIC-clade of the tribe Cichlasomatini, Musilová et al. 2008) distributed mostly in rivers of the Guyana shield, as well as in the Rio Negro basin, and the Amazon and Orinoco deltas. This group includes seven known species, four in the genus *Nannacara*, then two species recently extracted from *Nannacara* to the genus *Ivanacara* (Römer & Hahn, 2007), and the monotypic genus *Cleithracara*, which is basal to all the others. The cytogenetics of this clade remains poorly known since only two species of this group, *Cleithracara maronii* (Steindachner, 1881) with 2n=50 (Marescalchi 2004) and *Nannacara anomala* Regan, 1905 with 2n=44 (Thompson 1979) have been previously investigated.

In this study we present karyotypes and other chromosomal characteristics as revealed by CDD banding in five species of monophyletic clade of neotropical Cichlasomatine cichlids, namely *Cleithracara maronii*, *Ivanacara adoketa* (Kullander & Prada-Pedreros, 1993), *Nannacara anomala*, *Nannacara aureocephalus* Allgayer, 1983 and *Nannacara taenia* Regan, 1912. We further mapped the results onto the phylogenetic hypothesis from molecular analyses based on four genes. We discuss possible scenario of the karyotype evolution of the clade of dwarf cichlids within the tribe Cichlasomatini.

## Materials and methods

### Materials

The species included in the present study are listed in Table 1. Most of the individuals originated from aquarium trade from different breeders. Further, various collectors or ornamental-fish importers donated several samples for DNA analysis. Species were identified following Kullander and Nijssen (1989), Kullander and Prada-Pedreros (1993) and Staeck and Schindler (2004), and part of the analyzed fish was deposited in ICCU (Ichthyological Collection of Charles University, Prague). See Table 1 and Table 2.

### Cytogenetic analyses

Chromosomes were obtained by non-destructive isolation procedure from caudal fin regenerates as developed by Völker et al. (2006) and modified by Kalous et al. (2010). This method is based on regeneration of the caudal fin tissue after cutting a small part (2–3mm) from its margin. After five to seven days the regenerated tissue was cut and incubated in the solution with colchicine for two hours at room temperature. A few drops of fixative (methanol, acetic acid 3:1) were added to the tissue after this incubation and it was placed for 30min at 4°C. The tissue was washed twice in fixative, always staying for 30min at 4°C after the wash. Next, the tissue was placed into a drop of 20% acetic acid and gently mashed through a fine sieve. The suspension was dropped on a slide on a hot fig (45°C). After 20 seconds the drop of suspension was sucked up from the slide and dropped to a different place in the slide. Metaphase chromosomes were stained in 4% Giemsa solution in phosphate buffer (pH=7). Generally 5–50 metaphases per individual were evaluated. Chromosomes were classified according to Levan et al. (1964), to be consistent with most of the recent studies on cichlid fishes (Marescalchi 2004, Fedlberg et al. 2003, Poletto et al. 2010) and arranged to karyotypes by using ADOBE PHOTOSHOP, version CS7. The CDD fluorescent banding (Chromomycin A_3_/methyl green/DAPI) was performed following Mayr et al. (1985) and Sola et al. (1992).

### Molecular genetic analyses

DNA was extracted from the ethanol-preserved samples by the commercially available kits (QiaGen), and four target genes (cyt b, 16S rRNA, S7 first intron, RAG1) were amplified by PCR using primers according to Musilová et al. (2009). Sequences of the PCR products were obtained by commercial sequence-service company (Macrogen, South Korea, Netherlands). Sequences were aligned in BIO EDIT (Hall 1999) software and genes were concatenated for the bayesian analysis in MRBAYES 3.2. (Ronquist et al. 2012). Analysis parameters were: number of generations = 10,000,000, number of chains = 4, number of runs = 2, model set for every gene separately (and unlinked) based on the jModeltest (Posada 2008) results. Three additional species (*Bujurquina vittata, Aequidens metae* and *Laetacara thayeri*) from the same taxonomic tribus Cichlasomatini as *Nannacara + Ivanacara* were analyzed as well, and one species of the different tribus Geophagini (*Geophagus brasiliensis*) was determined as an outgroup for the phylogenetic analysis. Sequences were uploaded to GenBank (Table 1).

**Table 1.** Sample list for the present study. Details on individuals of cichlids investigated for the molecular genetics. Outgroup data were used from the original study (Musilová et al. 2008, 2009).

Individuals used in molecular phylogenetic analyses:			Accesion numbers in GenBank				Sample voucher
species	sample code	origin	cytb	16SrRNA	S7	RAG1	
***Geophagus brasiliensis***	outgroup – used from GenBank		EF470895	EU888080	EU199082	EU706360	-
***Bujurquina vittata***	outgroup – used from GenBank		EF432951	EF432892	EF432984	EU706385	-
***Aequidens metae***	outgroup – used from GenBank		EF432927	EF432882	EF432974	-	-
***Laetacara thayeri***	outgroup – used from GenBank		AY050608	EF432909	EF433001	EU706401	-
***Cleithracara maronii***	Cleith	aquarium trade	AY050614	EF432901	EF432993	EU706394	ICCU 0736
***Nannacara (Ivanacara) adoketa***	ADO	aquarium trade	EF432946	EF432903	EF432995	EU706396	ICCU 0745
***Nannacara (Ivanacara) adoketa***	In06	Rio Inirida	KJ136667	-	KJ136659	-	ICCU 1001
***Nannacara (Ivanacara) adoketa***	In03	Rio Inirida	KJ136668	-	KJ136660	-	ICCU 1002
***Nannacara anomala***	ANO	aquarium trade	AY050618	EF432898	EF432990	EU706391	ICCU 0746
***Nannacara anomala***	NaD	Orinoco delta	KJ136669	KJ136671	KJ136661	-	ICCU 1004
***Nannacara anomala* "Suriname**"	WSN	F1 progeny	-	-	KJ136654	-	-
***Nannacara aureocephalus* "blue**"	RNA01	aquarium trade	-	KJ136673	KJ136663	-	ICCU 1005
***Nannacara aureocephalus* "blue**"	RNA03	aquarium trade	-	KJ136674	KJ136664	-	-
***Nannacara aureocephalus* "blue**"	RNA04	aquarium trade	-	KJ136675	KJ136665	-	-
***Nannacara aureocephalus***	AUR	aquarium trade	EF432939	EF432899	EF432991	EU706392	ICCU 0747
***Nannacara* sp.**	SAR	import/unknown	-	KJ136670	KJ136655	KJ136666	ICCU 1003
***Nannacara prope aureocephalus* "brown**"	AurBrown01	aquarium trade	-	KJ136672	KJ136662	-	-
***Nannacara* sp. "Soumourou**"	NSP01	F1 progeny	-	-	KJ136656	-	-
***Nannacara* sp. "Oyapock**"	NSP02	F1 progeny	-	-	KJ136657	-	-
***Nannacara* sp. "Oyapock**"	NSP03	F1 progeny	-	-	KJ136658	-	-
***Nannacara* sp.**	AF045860	GenBank	-	AF045860	-	-	-
***Nannacara taenia***	TAE	aquarium trade	EF432921	EF432900	EF432921	EU706393	ICCU 0749

**Table 2.** Sample list for karyotypes analysis.

Individuals used in cytogenetic analyses (all from aquarium trade):		
Species	Number of analyzed individuals	Sex
*Cleithracara maronii*	3	undifferentiated
*Ivanacara adoketa*	3	2× male, 1× female
*Nannacara anomala*	5	3× male, 2× female
*Nannacara aureocephalus*	3	undiferentiated
*Nannacara taenia*	3	undiferentiated

## Results

### Karyotype characteristics

**Figure 1.**
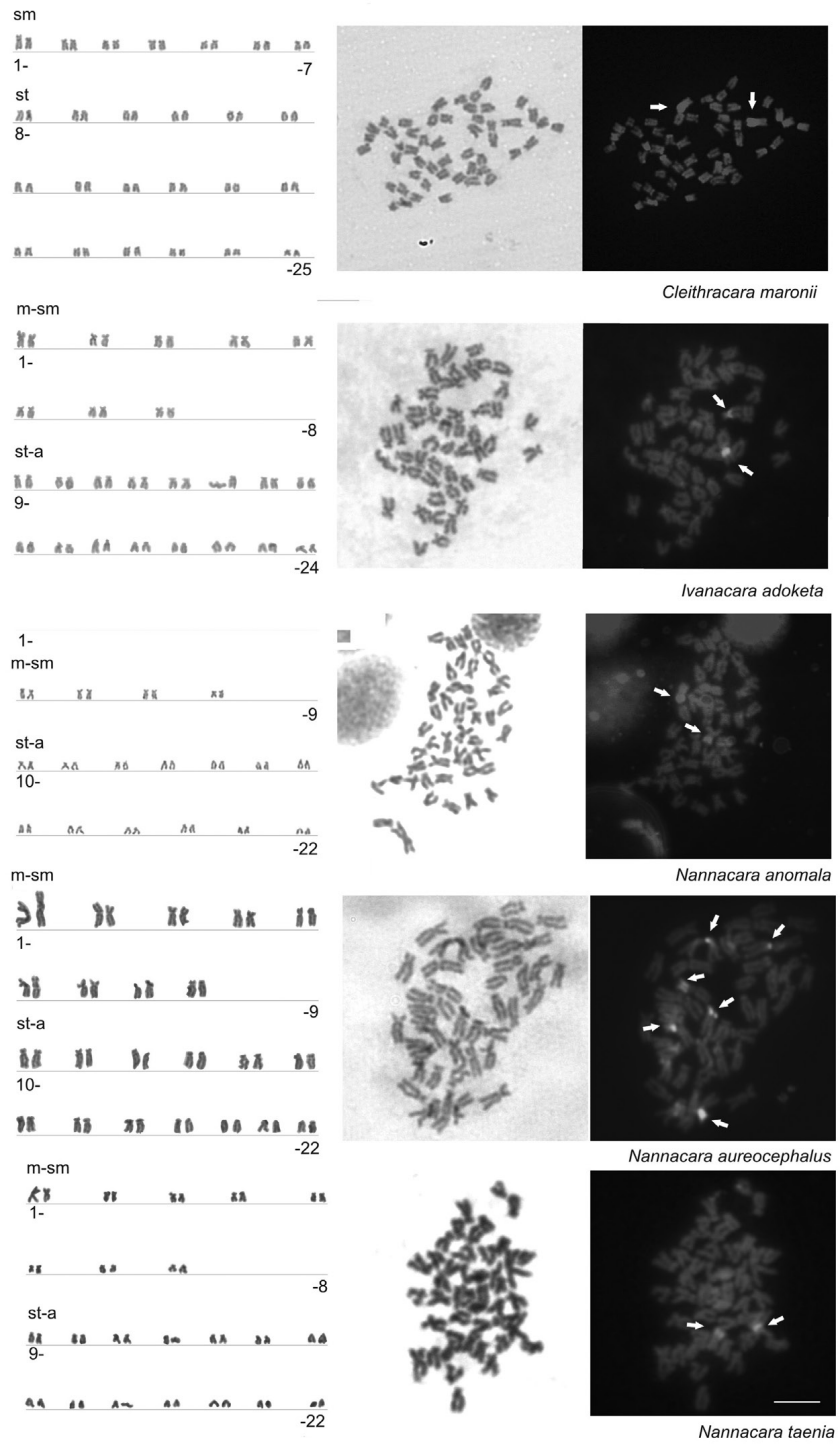
Karyotypes arranged from Giemsa stained chromosomes (left) of five species of cichlids: *Cleithracara maronii*, *Ivanacara adoketa*, *Nannacara anomala*, *Nannacara aureocephalus*, *Nannacara taenia*. Selected metaphases stained with Giemsa staining (center) and sequentially by CDD banding (right). White arrows indicate chromosomes with positive Chromomycin A_3_ signals. Bar=10µm.

Results are summarized in Fig. 1 and Table 3. Examined individuals of the species of genera *Nannacara, Ivanacara* and *Cleithracara* showed the diploid chromosome number 2n = 44 to 50 chromosomes. All three species of the genus *Nannacara* possessed 44 chromosomes and karyotype composed of 18 metacentric (m)-submetacentric (sm)+26 subtelocentric (st)-acrocentric (a) or 16m-sm+28st-a chromosomes, while *Ivanacara adoketa* had 2n = 48 and karyotype of 16m-sm+32st-a chromosomes, and *Cleihtracara maronii* had 2n = 50 composed of 14sm+36st-a chromosomes. Karyotypes of all studied species are shown in Fig. 1.

### CDD fluorescence

In the karyotypes of four studied species, namely *Cleithracara maronii*, *Ivanacara adoketa*, *Nannacara anomala*, and *Nannacara taenia*, the CMA_3_-positive signals were found on one chromosome pair, although probably not homologous in different species. In *Cleithracara maronii* the CMA_3_-positive signals were located on terminal parts of the largest m-sm chromosome pair, whereas in *Ivanacara adoketa* and *Nannacara taenia* the CMA_3_ signals were located a chromosome pair from st-a group, terminal parts in *Nannacara taenia* and around the centromere in *Ivanacara adoketa*. In *Nannacara anomala* the CMA_3_ signals were found on the terminal parts of a chromosome pair from m-sm group, but not on the largest pair. Contrarily, in the karyotype of *Nannacara aureocephalus*, the CMA_3_ signals were located on three m-sm chromosome pairs including the largest chromosome pair in the centromeric region. See Table 3 for more detail about the karyotype formulas and CMA_3_ phenotypes and Fig. 1 for representative metaphases and results of different staining steps.

### Phylogenetic analysis and karyotype differentiation

**Figure 2.**
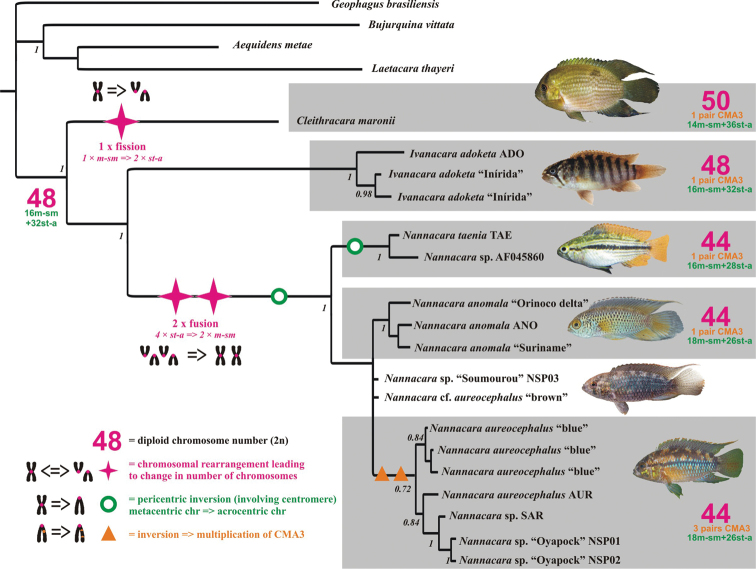
Phylogenetic relationships of cichlid fishes of genera *Nannacara*, *Ivanacara* and *Cleithracara*. Phylogenetic tree reconstructed based on the mitochondrial (cytochrome b, 16S rRNA) and nuclear (S7, RAG1) genes. Karyotype characteristics, such as diploid chromosomal number (2n), karyotype formula and CMA_3_ phenotype were mapped on the tree and interpreted under the most parsimonious criterion. Ancestral karyotype of the group evolved from the ancestral cichlid karyotype 48st-a (Mank and Avise 2006) by increasing number of sub-metacentric chromosomes. One fission (in *Cleithracara* clade) and two fusion events (in the *Nannacara* clade) were detected, followed by at least one pericentric inversion in the latter case causing the decrease of the number of sub-metacentric chromosomes. Second pericentric inversion occurred in *Nannacara taenia*, and another inversion leading to the multiplication of the CMA_3_ regions occurred in *Nannacara aureocephalus*.

Phylogenetic reconstruction based on the DNA sequences of up to four genes shows monophyly of the genus *Nannacara* (three species used in this study) and its sister relationship with the genus *Ivanacara* (one species present in our study). The monotypic genus *Cleithracara* (*Cleithracara maronii*) represents then basal lineage to the rest of *Nannacara* + *Ivanacara* (Fig. 2). The observed karyotype characteristics, i.e. the diploid chromosome number, the karyotype and the phenotype, were mapped on the phylogenetic tree and allowed reconstruction of the scenario of genome/karyotype evolution in the studied cichlids as well as to reconstruct as well as of the most likely hypothetical karyotype of an ancestor of the whole group. An ancestral karyotype of 2n = 48 was hypothesized as (16m-sm + 32 st-a) and was estimated as a basal stage for the clade by the most parsimonious reconstruction based on our material. The ancestor also had most likely only one pair of CMA_3_ sites (Fig. 2).

**Table 3.** Karyotype characteristics of the South American dwarf cichlids, including the diploid number of chromosomes (2n), chromosome categories, and CMA_3_ phenotype.

Species	2n	Karyotype	CMA_3_ signals
*Cleithracara maronii*	50	14sm+36st-a	1 sm pair
*Ivanacara adoketa*	48	16m-sm+32st-a	1 st-a pair
*Nannacara anomala*	44	18m-sm+26st-a	1 m-sm pair
*Nannacara aureocephalus*	44	18m-sm+26st-a	3 m-sm pair
*Nannacara taenia*	44	16m-sm+28st-a	1 st-a pair

## Discussion

### Cytogenetic characteristics

Two of the five species presented within this study have been previously studied in Thompson (1979), Marescalchi (2004) and reviewed in Feldberg et al. (2003). The karyotype of *Nannacara anomala* corresponds in both the chromosomal number (2n=44) and the karyotype (18m-sm+26st-a) to the results of Thompson (1979). The karyotype of *Cleithracara maronii* corresponds with various previous studies in chromosomal number (2n = 50; Marescalchi 2004, see Feldberg et al. 2003), but slightly differs in the karyotype description: while in our study we recognized seven pairs of sub-metacentric chromosomes (14m-sm+36st-a), Marescalchi (2004) found only six pairs of those. However, inspecting the study of Marescalchi (2004), we found one additional pair of sub-metacentric chromosomes in their original karyotype data as well, so it is fully comparable with our results.

In the clade of Neotropical cichlids, three trends in karyotype differentiation can be distinguished (Feldberg et al. 2003). First trend - also called Karyotype “A” by Thompson (1979) – is characterized by maintaining the ancestral karyotype of 2n=48 with mostly subtelocentric-acrocentric elements (karyotype of 48st-a, although not exclusively) and evolved mostly by the pericentric inversions (during which the centromere is shifted from the central position of chromosome). Second evolutionary trend is similar to the previous one and additionally suppose the chromosomal breakage/fission events (Feldberg et al. 2003), leading to the increasing diploid chromosome number usually to the 2n=50 or 52, extremely up to 2n=60). This karyotype is dominated by uniarmed chromosomes. The third evolutionary trend - also called Karyotype “B” in Thompson (1979) – is represented by the opposite evolutionary scenario - mostly centric fusions played role in evolution from the ancestral karyotype, which lead to reduction of diploid chromosome number accompanied by increasing number of metacentric and submetacentric chromosomes (Thompson 1979, Poletto et al. 2010). This trend of chromosome number reduction seems to be parallel to some other fish groups like it was uncovered in killifishes (Cyprinodontiformes, Nothobranchiidae) Völker et al. (2008).

All of the species within the studied evolutionary lineage have a higher proportion of sub-metacentric chromosomes in their karyotypes compared with the rest of cichlids (Poletto et al. 2010). Especially considering the fact that the ancestral cichlid karyotype has been postulated as 2n=48 and 48st-a, i.e. no sub-metacentric chromosomes are present (Poletto et al. 2010), the whole *Nannacara – Ivanacara – Cleithracara* clade seems to have evolutionary derived karyotype within cichlids. Based on Thompson’s (1979) classification, the whole lineage possess the karyotype type “B” characterized by higher proportion of the sub-metacentric chromosomes, although not all the species have the lower number of chromosomes then the ancestral stage, which is usually characteristic for the karyotype “B” as well (Thompson 1979). Interestingly, the chromosome rearrangements and formation of karyotype “B” occurred several times independently in cichlid evolution, as from 41 examined Neotropical cichlids, the karyotype “B” has been found in three unrelated lineages: in the species *Bujurquina vittata* (Heckel, 1840) (tribe Cichlasomatini), in the genus *Apistogramma* Regan, 1913 (tribe Geophagini) and in the genus *Symphysodon* Heckel, 1840 (tribe Heroini; sister tribe of Cichlasomatini; Thompson 1979). Strikingly, the most similar karyotype formula possessed by all the species of the genera *Apistogramma* (22-24m-sm+16-22st-a) and *Dicrossus* Steindachner, 1875 (12m-sm+34st-a), which also represent another two unrelated lineage of the dwarf cichlids (Thompson 1979, Feldberg et al. 2003), and then a few other species like *Cichlasoma paranaense* Kullander, 1983 (14-20m-sm+28-34st-a), *Mesonauta festivus/insignis* (Heckel, 1840) (12m-sm+36st-a), *Crenicichla niederleinii* (Holmberg, 1891) (14m-sm+34st-a) and *Astronotus ocellatus* (Agassiz, 1831) and *Astronotus crassipinnis* (Heckel, 1840) (12-18m-sm+30-36st-a, Feldberg et al. 2003). Note, that although the karyotype composed of mostly subtelocentric-acrocentric chromosomes is considered as ancestral for the cichlids, it is not generally ancestral trait for other fish groups. Therefore, the emergence of karyotype “B” (with more sub-metacentric chromosomes) probably represents secondary change back to the “common teleost karyotype” (Thompson 1979, Arai 2011).

### CMA_3_ patterns

The CMA_3_ signals represent usually the GC-rich DNA segments of heterochromatic regions, often correlated with the location of active or inactive NORs, usually represented by the rDNA regions in genome (Schmid and Guttenbach 1988, Ráb et al. 1999, Poletto et al. 2010, but see Fontana et al. 2001, Gromicho et al. 2005 or Saitoh and Laemmli 1994). The number of CMA_3_ signals found within this study corresponds to what has been previously observed in cichlids – i.e. the most common number of NORs in Neotropical cichlids is one pair, but in some species were found up to three pairs (Feldberg et al. 2003, Poletto et al. 2010). In the *Nannacara – Ivanacara – Cleithracara* clade, all species except for *Nannacara aureocephalus* possess only one pair of CMA_3_ signals in their karyotype. *Nannacara aureocephalus* has three pairs of CMA_3_ signals, which is usually interpreted as the result of inversion followed by the multiplication of the rDNA regions (Poletto et al. 2010). Further, one of the observed CMA_3_ regions in this species is located in the centromeric region.

After Feldberg et al. (2003), one pair of NORs on the larger pair of chromosomes represents the most common NOR phenotype for the whole family Cichlidae. Further, Hsu et al. (1975) suggested that species with the single pair of NORs should be considered as more primitive that the karyotype with several NOR pairs hinting that the ancestral karyotypes possess less NORs than the evolutionary derived. Multiplication of NORs is usually caused by the chromosomal rearrangements, such as translocation or inversion but recently an increasing number of studies has shown the cases of rDNA multiplication caused by the activity of transposable elements.(Cioffi et al. 2010, Symonová et al. 2013, Schneider et al. 2013). As summarized in Feldberg et al. (2003), five out of 15 analysed species of the subfamily Cichlasomatinae (tribes Heroini + Cichlasomatini) possess multiple NOR pairs, i.e. *Caquetaia spectabilis* (Steindachner, 1875) (Feldberg et al. 2003), *Cichlasoma paranaense* Kullander, 1983 (Feldberg et al. 2003), *Mesonauata insignis* and *Mesonauata festivus* (Heckel, 1840) (Feldberg et al. 2003) and *Symphysodon aequifasciatus* Pellegrin, 1904 (Feldberg et al. 2003).

### Phylogeny of *Nannacara* – *Ivanacara* – *Cleithracara* cichlids

The phylogenetic reconstruction of the *Nannacara – Ivanacara – Cleithracara* clade (also called NIC clade in Musilová et al. 2008, 2009) corresponds to the results observed in the previous studies (Musilová et al. 2008, 2009). This suggests the basal position of the monotypic genus *Cleithracara* followed by the *Ivanacara* (one species) sister to the rest of fishes from the genus *Nannacara* (three species). Within *Nannacara*, the *Nannacara taenia* has basal position and *Nannacara anomala* + *Nannacara aureocephalus* represent the sister species. In this study, we did not include two species of the studied clade, i.e. *Nannacara quadrispinae* and *Ivanacara bimaculata*, which we failed to obtain either as live individuals for cytogenetics, or as samples for DNA analysis. Especially *Ivanacara bimaculata* would be crucial for confirmation of monophyly of the genus *Ivanacara*, since *Ivanacara bimaculata* was previously found as closely related to the fishes of the genus *Nannacara* then to *Ivanacara adoketa* based on morphological data set (Musilová et al. 2009).

Within *Nannacara aureocephalus*, more distinct forms are known; some of them were introduced into the aquarium trade under different names. So far no robust revision of *Nannacara* is available, and it is therefore difficult to make any taxonomic conclusion based on our data set. However, at least two different forms of *Nannacara aureocephalus* are spread among the aquarium hobbyist within Central Europe (Germany, Poland, Czech Republic, Slovakia) – one of them called “blue” and the other one called “brown” both included in our analyses. These forms are not of artificial origin, as usually F1 progeny of the wild caught individuals has been studied. Intuitively, the blue morph shows more light-blue coloration with iridescent elements both on the face and body, while the “brown” form doesn’t have the iridescent coloration and possess darker brown to dark-green coloration pattern. We have shown that those two morphs are genetically distinct; however, more detailed future work is necessary on this species/genus.

### Karyotype differentiation

Cichlid karyotypes show some general common features - for example many species from African and Neotropical cichlids possess one pair of significantly larger chromosomes. Although the homology of the largest chromosome within the African lineage has been proved (Ferreira et al. 2010) as well as high synteny conservation of African cichlid genomes (Mazzuchelli et al. 2012), it is, however, not yet clear to what extent is the homology present across the whole family Cichlidae (Valente et al. 2009).

Although all the studied species from the *Nannacara – Ivanacara – Cleithracara* clade are characterized by the karyotype “B” (Thompson 1979), they underwent different evolutionary paths in past. The phylogenetic reconstruction of the karyotype evolution suggests the following scenario: from the ancestral karyotype, first the karyotype of the *Cleithracara maronii* (2n = 50; 14mt-sm + 36 st-a) evolved by fission event of one sub-metacentric chromosome pair, falling apart into two additional pairs of subtelocentric-acrocentric chromosomes. While the karyotype of *Ivanacara adoketa* remained unchanged compared with the ancestral one, in the lineage of *Nannacara*, two fusions occurred decreasing chromosomal number to 2n = 44. These fusions were followed by pericentric inversions, which again decreased the number of sub-metacentric chromosomes. At least one pericentric inversion happened in the base of all *Nannacara*, and additional pericentric inversion happened in the *Nannacara taenia* lineage. Finally, two inversion impacting CMA_3_ regions happened in *Nannacara aureocephalus* leading to the multiplicaiton of these signals.

The proposed mechanisms of chromosomal rearrangements are described in cichlids as well as in other fish species. Usually the sub-metacentric chromosome arises during the (centric) fusion, when two acrocentric-telocentric chromosomes fuse (Thompson 1979). However, the number of sub-metacentric chromosomes in karyotype is not evolutionarily stable. The sub-metacentric chromosome changes back to the acrocentric-subtelocentric chromosome by inversion, which involves the centromere, i.e. the pericentric inversion (Feldberg et al. 2003, Poletto et al. 2010). Further, those pericentric inversions are considered as the main mechanism generally contributing to changes in chromosome arms size in various percomorph lineages (Galetti et al. 2000, Affonso 2005). In general, the taxon sampling within such comparative studies is however still too low to be able to make a strong conclusion about the general trends in cichlid karyotype evolution (Feldberg et al. 2003, Poletto et al. 2010).

To conclude, we aimed to provide a comparative study on a small scale of three genera combining molecular and cytogenetic approaches. Assuming that cytogenetic data provide additional information, which is undetectable by molecular genetics (Ráb et al. 2007), we expected a broad insight into the genome evolution of the studied group. In the dwarf cichlid genus *Nannacara* and its relatives (*Ivanacara* and *Cleithracara*), we reconstructed the phylogeny and we found substantial amount of karyotype characteristics, which we were able to interpret in the evolutionary context.

## References

[B1] Affonso PR Galetti PM Jr (2005) Chromosomal diversification of reef fishes from genus *Centropyge* (Perciformes, Pomacanthidae). Genetica 123(3): 227–233. doi: 10.1007/s10709-004-3214-x 15954493 10.1007/s10709-004-3214-x

[B2] Arai R (2011) Fish Karyotypes. Springer, Japan, 340 pp. doi: 10.1007/978-4-431-53877-6

[B3] Cioffi MB Martins C Bertollo LA (2010) Chromosome spreading of associated transposable elements and ribosomal DNA in the fish *Erythrinus erythrinus*. Implications for genome change and karyoevolution in fish. BMC Evolutionary Biology 10: 271–280. doi: 10.1186/1471-2148-10-271 20815941 10.1186/1471-2148-10-271 PMC2944182

[B4] Eschmeyer WN Fricke R (2012) Catalog of Fishes electronic version. Available from http://research.calacademy.org/research/ichthyology/catalog/fishcatmain.asp

[B5] Feldberg E Bertollo LAC (1985) Karyotypes of 10 species of Neotropical cichlids (Pisces, Perciformes). Caryologia 38(3–4): 257–268. doi: 10.1080/00087114.1985.10797749

[B6] Feldberg E Porto JIR Bertollo LAC (2003) Chromosomal changes and adaption of cichlid fishes during evolution. In: Val AL Kapoor BG (Eds) Fish Adaption.Science Publishers, Enfield-NH, USA, 285–308

[B7] Ferreira I Poletto B Kocher TD Mota-Velasco JC Penman DJ Martins C (2010) Chromosome evolution in African cichlid fish: contributions from the physical mapping of repeated DNAs. Cytogenetic and genome research 129(4): 314–22. doi: 10.1159/000315895 20606399 10.1159/000315895 PMC3202915

[B8] Fontana F Tagliavini J Congiu L (2001) Sturgeon genetics and cytogenetics: recent advancements and perspectives. Genetica 111: 359–373. doi: 10.1023/A:1013711919443 11841180 10.1023/a:1013711919443

[B9] Galetti PM Jr Aguilar CT Molina WF (2000) An overview on marine fish cytogenetics. Hydrobilogia 420: 55–62. doi: 10.1007/978-94-017-2184-4_6

[B10] Gromicho M Ozouf-Costaz C Collares-Pereira MJ (2005) Lack of correspondence between CMA3-, Ag-positive signals and 28S rDNA loci in two Iberian minnows (Teleostei, Cyprinidae) evidenced by sequential banding. Cytogenetic Genome Research 109: 507–511. doi: 10.1159/000084211 15905646 10.1159/000084211

[B11] Hall TA (1999) BioEdit: a user-friendly biological sequence alignment editor and analysis program for Windows 95/98/NT. Nucleic Acids Symposium Series 41: 95–98

[B12] Hsu TC Spirito SE Pardue LM (1975) Distribution of 18/28S ribosomal genes in Mammalian genomes. Chromosoma 53: 25–36. doi: 10.1007/BF00329388 1104290 10.1007/BF00329388

[B13] Kalous L Knytl M Krajáková L (2010) Usage of non-destructive method of chromosome preparation applied on silver Prussian carp (*Carassius gibelio*). In: Kubík S Barták M (Eds) Proceedings of the Workshop on Animal Biodiversity.Jevany, July 7, 2010, 57–60

[B14] Kocher TD (2004) Adaptive evolution and explosive speciation: the cichlid fish model. Nature Reviews Genetics 5: 288–98. doi: 10.1038/nrgl316 10.1038/nrg1316 15131652

[B15] Kornfield IL (1984) Descriptive Genetics of Cichlid fishes. In: Turner BJ (Ed) Evolutionary Genetics of Fishes.Plenum Press, New York, 591–616. doi: 10.1007/978-1-4684-4652-4_12

[B16] Kullander SO (1998) A phylogeny and classification of the South American Cichlidae (Teleostei: Perciformes). In: Malabara LR Reis RE Vari RP Lucena ZM Lucena CAS (Eds) Phylogeny and Classification of Neotropical Fishes, Part 5.EDUPUCRS, Porto Alegre, 461–498

[B17] Kullander SO Nijssen H (1989) The Cichlids of Surinam. E. J. Brill, Leiden, 251 pp

[B18] Kullander SO Prada-Pedreros S (1993) *Nannacara adoketa*, a new species of cichlid fish from the Rio Negro in Brazil Ichthyological Exploration of Freshwaters 4(4): 357–366.

[B19] Levan A Fredga K Sanger AA (1964) Nomenclature for centromeric position on chromosomes. Hereditas 52: 201–220. doi: 10.1111/j.1601-5223.1964.tb01953.x

[B20] Mank, JE Avise JC (2006) Phylogenetic conservation of chromosome numbers in Actinopterygiian fishes. Genetica 127: 321–327. doi: 10.1007/s10709-005-524 16850236 10.1007/s10709-005-5248-0

[B21] Marescalchi O (2004) Karyotype and mitochondrial 16S gene characterizations in seven South American Cichlasomatini species (Perciformes, Cichlidae). Journal of Zoological Systematics & Evolutionary Research 43: 22–28. doi: 10.1111/j.1439-0469.2004.00285.x

[B22] Mayr B Ráb P Kalat M (1985) Localisation of NORs and counterstain-enhanced fluorescence studies in *Perca fluviatilis* (Pisces, Percidae). Genetica 67: 51–56. doi: 10.1007/BF02424460

[B23] Mazzuchelli J Kocher TD Yang F Martins C (2012) Integrating cytogenetics and genomics in comparative evolutionary studies of cichlid fish. BMC genomics 13(1): 463–477. doi: 10.1186/1471-2164-13-463 22958299 10.1186/1471-2164-13-463 PMC3463429

[B24] Musilová Z Říčan O Janko K Novák J (2008) Molecular phylogeny and biogeography of the Neotropical cichlid fish tribe Cichlasomatini (Teleostei: Cichlidae: Cichlasomatinae). Molecular Phylogenetics and Evolution 46(2): 659–72. doi: 10.1016/j.ympev.2007.10.011 18055225 10.1016/j.ympev.2007.10.011

[B25] Musilová Z Říčan O Novák J (2009) Phylogeny of the Neotropical cichlid fish tribe Cichlasomatini (Teleostei: Cichlidae) based on morphological and molecular data, with the description of a new genus. Journal of Zoological Systematics and Evolutionary Research 47(3): 234–247. doi: 10.1111/j.1439-0469.2009.00528.x

[B26] Nakatani Y Takeda H Kohara Y Morishita S (2007) Reconstruction of the vertebrate ancestral genome reveals dynamic genome reorganization in early vertebrates. Genome Research 17(9): 1254–1265. doi: 10.1101/gr.6316407 17652425 10.1101/gr.6316407 PMC1950894

[B27] Ohno S Muramoto J Klein J Atkin NB (1969) Diploid-tetraploid relationship in clupeoid and salmon fish. Chromozómes Today 2: 139–147

[B28] Poletto AB Ferreira IA Cabral de Mello DC Nakajima RT Mazzuchelli J Ribeiro HB Venere PC Nirchio M Kocher TD Martins C (2010) Chromosome differentiation patterns during cichlid fish evolution. BMC Genetics 11: 50. doi: 10.1186/1471-2156-13-2 10.1186/1471-2156-11-50 PMC2896337 20550671

[B29] Posada D (2008) jModelTest: Phylogenetic Model Averaging. Molecular Phylogenetics Evolution 25: 1253–1256. doi: 10.1093/molbev/msn083 10.1093/molbev/msn083 18397919

[B30] Ráb P Bohlen J Rábová M Flajšhans M Kalous L (2007) Cytogenetics as a tool in fish conservation: the present situation in Europe. In: Pisano E, Ozouf- Costaz C, Foresti F, Kapoor BG (Eds) Fish Cytogenetics. Science Publishers, Enfield, USA.

[B31] Ráb P Rábova M Reed KM Phillips RB (1999) Chromosomal characteristics of ribosomal DNA in the primitive semionotiform fish, longnose gar *Lepisosteus osseus*. Chromosome Research 7: 475–480. doi: 10.1023/A:1009202030456 10560970 10.1023/a:1009202030456

[B32] Römer U Hahn I (2007) *Ivanacara* gen. n. (Teleostei: Perciformes, Cichlasomatini) – a new genus of cichlids from the Neotropis. In: Römer U (Ed) Cichlid Atlas.Volume 2, Natural History of South American Dwarf Cichlids, Part 2. Mergus Verlag GmbH, Melle, 1190–1197

[B33] Ronquist F Teslenko M Van der Mark P Ayres, DL Darling A Höhna S Larget B Liu L Suchard MA Huelsenbeck JP (2012) MrBayes 3.2: efficient Bayesian phylogenetic inference and model choice across a large model space. Systematic Biology 61(3): 539–42. doi: 10.1093/sysbio/sys029 22357727 10.1093/sysbio/sys029 PMC3329765

[B34] Saitoh Y Laemmli UK (1994) Metaphase chromosome structure: bands arise from a differential folding path of the highly AT-rich scaffold. Cell 76: 609–622. doi: 10.1016/0092-8674(94)90502-9 7510215 10.1016/0092-8674(94)90502-9

[B35] Schmid M Guttenbach M (1988) Evolutionary diversity of reverse (R) fluorescent chromosome bands in vertebrates. Chromosoma 97: 327–344. doi: 10.1007/BF00327367 10.1007/BF00327367 2976364

[B36] Schneider CH Gross MC Terencio ML do Carmo EJ Martins C Feldberg E (2013) Evolutionary dynamics of retrotransposable elements Rex1, Rex3 and Rex6 in neotropical cichlid genomes. BMC Evolutionary Biology 13: 152. doi: 10.1186/1471-2148-13-152 10.1186/1471-2148-13-152 PMC3728117 23865932

[B37] Sola L Rossi AR Laselli V Rasch, EM Monaco PJ (1992) Cytogenetics of bisexual/unisexual species of Poecilia. II. Analysis of heterochromatin and nucleolar organizer regions in Poecilia mexicana mexicana by C-banding and DAPI, quinacrine, chromomycin A3, and silver staining. Cytogenetics and Cell Genetics 60: 229–235. doi: 10.1159/000133346 1380417 10.1159/000133346

[B38] Staeck W Schindler I (2004) *Nannacara quadrispinae* sp. n. – a new dwarf cichlid fish (Teleostei: Perciformes: Cichlidae) from the drainage of the Orinoco Delta in Venezuela. Zoolgishe Abhandlungen aus dem Staatlichen Museum fur Tierkunde in Dresden 54: 155–161

[B39] Symonová R Majtánová Z Sember A Staaks GBO Bohlen J Freyhof J Rábová M Ráb P (2013) Genome differentiation in a species pair of coregonine fishes: an extremely rapid speciation driven by stress-activated retrotransposons mediating extensive ribosomal DNA multiplications. BMC Evolutionary Biology 3: 42. doi: 10.1186/1471-2148-13-42 10.1186/1471-2148-13-42 PMC3585787 23410024

[B40] Thompson KW (1979) Cytotaxonomy of 41 species of Neotropical Cichlidae. Copeia 4: 679–691. doi: 10.2307/1443877

[B41] Valente GT Schneider CH Gross MC Feldberg E Martins C (2009) Comparative cytogenetics of cichlid fishes through genomic in-situ hybridization (GISH) with emphasis on Oreochromis niloticus. Chromosome Research 17(6): 791–9. doi: 10.1007/s10577-009-9067-5 19685270 10.1007/s10577-009-9067-5

[B42] Völker M Sonnenberg R Ráb P Kullmann H (2006) Karyotype differentiation in Chromaphyosemion killifishes (Cyprinodontiformes, Nothobranchiidae). II: Cytogenetic and mitochondrial DNA analyses demonstrate karyotype differentiation and its evolutionary directionin C. riggenbachi. Cytogenetic Genome Research 115: 70–83. doi: 10.1159/000094803 16974086 10.1159/000094803

[B43] Völker M Ráb P Kullmann H (2008) Karyotype differentiation in Chromaphyosemion killifishes (Cyprinodontiformes, Nothobranchiidae): patterns, mechanisms, and evolutionary implications. Biological Journal of the Linnean Society 94(1): 143–153. doi: 10.1111/j.1095-8312.2008.00967.x

